# Articulating biological and social approaches in child and adolescent psychiatry

**DOI:** 10.3389/frcha.2022.1065932

**Published:** 2022-12-21

**Authors:** Sélim Benjamin Guessoum, Laelia Benoit, Isaiah Thomas, Jasmina Mallet, Jordan Sibeoni, Cyril Hanin, Marie Rose Moro

**Affiliations:** ^1^ Université Paris Cité, PCPP, Paris, France; ^2^ Department of Child and Adolescent Psychiatry, AP-HP, Cochin University Hospital, Paris, France; ^3^ Université Paris-Saclay, Inserm U1018, CESP, Team DevPsy, Villejuif, France; ^4^ Yale School of Medicine, Child Study Center, New Haven, CT, United States; ^5^ Université Paris Cité, Inserm U1266, Institute of Psychiatry and Neuroscience of Paris, Paris, France; ^6^ Department of Psychiatry, AP-HP, Louis Mourier University Hospital, Colombes, France; ^7^ Centre Hospitalier d’Argenteuil, Department of Child and Adolescent Psychiatry, Argenteuil, France; ^8^ Université Paris Cité, Inserm U1153, ECSTRRA Team, Paris, France; ^9^ Centre de Référence des Maladies Rares à Expression Psychiatrique & PSYDEV Team, Department of Child and Adolescent Psychiatry, Pitié-Salpêtrière University Hospital, AP-HP, Sorbonne University, Paris, France

**Keywords:** child and adolescent psychiatry, transcultural psychiatry, biological psychiatry, social psychiatry, pluralistic, integrative, complementary, research design

## Abstract

Child and adolescent psychiatry has been based on numerous fields of research and theories, including neuroscience, physiology, psychology (developmental, psychodynamic, systemic, cognitive-behavioral, etc.), anthropology, sociology, and education sciences. Integrating transdisciplinary knowledge in multi-level models is an ongoing challenge for the future that is not immediately applicable in clinical practice and research. Articulating, i.e., to connect, to be jointed, (psycho)biological and (psycho)social approaches in child and adolescent psychiatry is a daily challenge for clinicians and researchers. Research is often limited to specific fields whereas real-life clinical practice needs a pluralistic approach. Research designs, tools, and clinical training need to provide knowledge applicable to the necessarily pluralistic daily clinical practice. This article provides some perspectives on how to articulate biological and social approaches, from research to clinical practice, and discusses the concept of pluralistic approaches, multimodal interventions, and how to provide articulated mental health care and training. Suggestions to better articulate biological and social approaches are provided: (I) State that the research object can be approached from different theoretical, research and clinical angles and explain the one chosen; (II) Propose synthesis articles that articulate biological and social knowledge; (III) Design biological studies that take into account social factors, and design social studies that take into account biological factors; (IV) Design transcultural tools; (V) Build pluralistic interventions, i.e., therapeutic modalities and mental health care settings that articulate biological and social approaches; (VII) Develop training in pluralistic articulated care.

## Introduction

Child and adolescent psychiatry (CAP) aims to treat disorders of thinking, feeling, and/or behavior affecting children, adolescents, and their families. Articulating biological and social approaches is necessary to grapple with the complexities of patients and mental disorders. As examples of this tension between biological and social approaches, one can cite the three following: (1) Possession by spirits in an adolescent from a Islamic culture could be a culturally meaningful non-psychotic syndrome or a psychotic disorder or both could be interrelated with one another; such complexities can lead to misdiagnosis; (2) Anorexia nervosa has clear neurobiological correlates, including a strong genetic component, but family therapy, which focuses on environmental, familial, and social factors, is an evidence-based treatment (1–3); (3) Autism spectrum disorder has been progressively explained through in-depth knowledge at the genomic, molecular, cellular, and neural-circuit levels, yet interventions focus on improving interactions and communication between patients and their families(4, 5). Further examples are detailed in Table 1.

**Table 1:** Examples.

**A. An illustration *via* a common case of psychosis** A 14-year-old boy from Morocco is diagnosed with schizophrenia characterized by a delusion of spirit possession (djinn); the family does not accept/support antipsychotic treatment.	
**Biological approach**	- Genetic, molecular, and neurologic impairments are found in schizophrenia (6) - Research on the specific etiology through biological and neurological testing (7) - Antipsychotic treatment (8)
**Social approach**	- Imigration is a risk factor for schizophrenia (9) - Possession by a djinn is a common cultural etiology *(i.e., cultural representation of the cause)* of positive symptoms (10) - Family and cultural representation and narration of the disease are central in the experience of disease and adherence to care (11, 12)
**Expectations of a pluralistic approach**	- Addressing patient, family and cultural narration supports the therapeutic alliance, adherence to care, and collaboration with care providers, and ensures ethical care - A biological approach is necessary in schizophrenia but ineffective (because inapplicable) without family and patient adherence
**B. An illustration *via* autism spectrum disorder**	
**Biological approach**	- Knowledge of genomic, molecular, cellular and neural-circuit impairments (4) - Pharmacologic treatments are useful [e.g., melatonin levels are reduced in autism, and providing melatonin may improve sleep and daytime behavior (13–15)
**Social approach**	- Interventions focus on interactions and communication with patients and their families (5) - Cultural and social factors influence community beliefs and families’ understandings of autism (16)
**Expectations of a pluralistic approach**	- Cultural and contextual adaptations are required and need to be studied to make interventions feasible, acceptable, and effective in non-Western countries, e.g., Sub-Saharan Africa (17) - The clinical management of autism, a biological disorder, is embedded in parent-child interactions, which vary across cultures (18)
**C. An illustration *via* anorexia nervosa**	
**Biological approach**	- Eating disorders have important genetic and biological correlates (1, 19) - Antipsychotic treatments may have a therapeutic effect in anorexia nervosa, but data are weak (20–22) - Biological hypotheses on anorexia nervosa lead to new therapeutic options such as neuromodulation (20, 23)
**Social approach**	- Family-based therapy is a first-line treatment (2) - Social factors vary over time and influence body shape preferences and the epidemiology of eating disorders (24)
**Expectations of a pluralistic approach**	- Anorexia nervosa is a severe condition requiring multiple interventions (e.g. family interventions, medication, and nutritional rehabilitation) - Timing of interventions may influence paients’ outcome, a pluralistic reflexion on interventions timing is needed (24)

Child and adolescent psychiatry has been based on numerous fields of research and theories, including neuroscience, genetics, physiology, psychology (developmental, psychodynamic, systemic, cognitive-behavioral, etc.), anthropology, sociology, and education sciences. Over the past 70 years, we have witnessed the demise of “big” theories in CAP purporting to offer overarching explanations and in its place the development of specialized research fields that provide strategies for tackling particular questions (25). Leon Eisenberg, a prominent U.S. child psychiatrist and pioneer in autism and ADHD research, criticized knowledge silos in CAP as leading to ineffective and even harmful interventions. In a dramatic but meaningful formulation, Eisenberg described the shift from overly psychoanalytic to overly biological psychiatry during the second half of the 20th century as a switch from “brainless” to “mindless” psychiatry (26). Various fields in CAP are evolving simultaneously, with distinct theories, concepts, representations, and interventions. In this article, we schematize the diversity of approaches into (psycho)biological approaches vs. (psycho)social approaches. Biological approaches refer to the conception of psychiatry as a medical science based on biology, physiology and neuroscience. This perspective posits that biology grounds medical practice and that other sciences can contribute but cannot displace biology from its central role (27). Biological approaches have brought important changes to CAP, such as the vast body of research on the use of stimulants to treat ADHD (25). Nevertheless, social approaches, such as sociology and anthropology, are critical in CAP: These approaches are grounded in the concept that humans are fundamentally social beings, and that social factors are key determinants of health (28). Thus, in the context of CAP, the child is not only an individual and must be viewed within a larger familial, social, and cultural context (29).

Evidence-based medicine (EBM) has influenced research methods in the last few decades, promoting quantitative research, which tends to exclude cultural factors, and thus minority groups, in order to obtain homogeneous study populations. Consequently, there is scarce transcultural data in CAP (30, 31). This lack of research knowledge and the need for updated and integrative diagnostic and interventional models remains a challenge in the field. A pluralistic approach argues that multiple independent methods are necessary in the understanding and treatment of mental illness and that no single method is sufficient (32).

There are numerous obstacles that impede the implementation of a pluralistic approach in CAP: the difficulty of identifying and treating diseases in CAP; objects in CAP are multiple and heterogeneous [e.g., Kleinman's developments on the personal and social meanings disease, illness, and sickness (33, 34)]; the divide between biologically oriented child psychiatrists and other types of child psychiatrists (35); the lack of unifying theories and the pragmatic oversimplification for the sake of practice and training (36). Integrative transdisciplinary models in research are promising, but they are not immediately applicable to clinical practice [e.g., translational social neuroscience (37); cultural neuroscience (38); neurophenomenology (39)]. In our opinion, significant gaps persist between biological and social approaches, and between theory, research, and clinical practice. The objective of this article is to provide practical perspectives on how to articulate (i.e., *to connect, to be jointed*) biological and social approaches in CAP.

## Definitions: pluralistic articulated approach

Biological and social approaches have not only different theories and concepts (self, disorders, emotions, etc.), but also different methodologies. Biological research is more often *quantitative* and *nomothetic*, i.e., it provides standardized, generalizable knowledge on disorders. Social research is often *qualitative* and *idiographic*, i.e., it provides *in-depth* knowledge on the experience of patients (40, 41)*,* though numerous nomothetic studies also exist in the social sciences*. Nomothetic* methods do not take into account the personal and subjective factors involved in patients' disorders. In contrast, *idiographic* methods, based on the patient and his or her particular features, can help identify the social experience of mental disorders.

We provide brief definitions of some important concepts in the Supplementary materials, and we highlight three of them here: pluralistic, complementary and integrative. A pluralistic approach states that multiple independent methods are necessary in the understanding and treatment of mental illness and that no single method is sufficient (32). In pluralistic approaches, the conceptual differences are assumed to understand mental and brain phenomena (32). Care providers can combine actions from different fields and theories, wondering which ones are relevant in a given case (rather than applying a little of each for every disorder). Complementary frames of reference (*complémentarisme*) is a concept historically based on George Devereux's French school of transcultural psychiatry: it argues for the necessary *non-simultaneous* use of several methods (at the time, anthropology and psychoanalysis) (42). Integrative psychiatry aims at providing a synthetic approach to the distinct psychiatry fields (41). In this article, we use the term *pluralistic approach* to refer to the use of multiple independent methods, articulated rationally and non-simultaneously but with possible overlap, in accordance with both evidence-based and patient-centered medicine.

There are essentially two options: (1) To assume a pluralistic approach with separated concepts and research methods, or (2) To build models whose theories encompass the traditionally separated fields. With the first option, one can study a single theme with two or more methods *via* distinct research protocols. For example, one can perform, on the one hand, biological studies on adolescent depression and, on the other hand, social research on this topic (43, 44). The objects, methods, and theories would be distinct between these methods. As another example, within a single team, in adult psychiatry, we performed, on the one hand, a quantitative epidemiological study and, on the other hand, a qualitative psychological study on care providers' mental health during the COVID-19 pandemic (45, 46). The second option, integrating typically separate fields, requires a wider and deeper theoretical foundation. Achieving this goal would be, in our opinion, the most ambitious and important transformation for the future of psychiatry (47). Like biological processes, social factors influence the brain's development and functioning. For example, children in situations of severe social isolation demonstrate abnormal brain development and language impairments (48). Proposing sociobiological models could constitute the future of CAP (49). Until then, clinicians and researchers still require perspectives to grapple with the coexistence of social and biological approaches.

## Research: articulating biological and social approaches from clinical description to research

The articulation of social and biological approaches should be carried out starting from the clinical description phase. Otherwise, if psychiatrists follow a standard descriptive criteriologic approach, only a part of the patients' experience—the objective, standardized, generalizable and observable aspects—will be reported and thus shape the research perspectives. Additionally, the expression of mental distress varies within populations and across cultures, depending on social and biological factors, and is also characterized by distinct cultural presentations that demand a social assessment by psychiatrists [e.g., *hikikomori* syndrome in adolescents (50)].

Collecting and synthesizing both sociocultural knowledge and neurobiological knowledge on a subject is not an obvious approach, but we argue that it should be more widely utilized. We applied this synthetic approach to study clinical lycanthropy, a syndrome in which patients have the delusional belief that he or she is turning into a wolf (51, 52). We conducted a systematic review of medical publications and linked together biological and cultural knowledge (53). The reader thus witnesses the clear necessity of both approaches: clinical lycanthropy is related to neuropsychiatric disorders, but it occurs in a particular environmental and cultural context. Consequently, we hope that any reader on this topic will have a representation of clinical lycanthropy that integrates both approaches. Next steps consist of offering multilevel perspectives that include the brain and its environment (47).

We offer the following suggestions:

• [I] All available sociocultural and biological data should be included in clinical descriptions and case reports. Psychiatrists should exhibit the same precision in describing both the biological (neurological) and social features of disease as early psychiatrists did in their meticulous clinical observations;
• [II] Biological studies should consider the social context, and social studies should consider the biological context;
• [III] Scientific syntheses (e.g., reviews) should gather both biological and social data.

## Tools: build tools that meet the needs of pluralism

Pertinent validated tools (e.g., questionnaires and scales), designed for research or clinical practice, are keystones to articulating biological and social approaches. Instruments are scarce in CAP, and their use is complicated by the dynamic aspect of development and the necessary adaptation of instruments to the age and developmental stage of each patient. Standard rating scales can reflect a nomothetic representation of the measured variables, whereas transcultural validity includes an idiographic perspective.

The tools available to us shape research designs and clinicians’ representations, notably by capturing some cultural forms of mental health experiences, while rendering others invisible. In standard evidence-based and nomothetic CAP approaches, there is a lack of culturally appropriate and transculturally valid instruments (54, 55). Some Western-derived scales distort or miss some culturally specific dimensions of mental distress, inducing a form of *experience measure fallacy*, or an error in measuring the experience of patients by superimposing Western experiences of mental distress (as a parallel to Kleinman's *category fallacy*, referring the application of a nosological category developed for a particular cultural group to the members of another culture for whom it lacks coherence and validity has not been established) (56–58). A Western scale in adult psychiatry would miss a culturally meaningful form of dysphoria that the Afghan symptom checklist does capture: *jigar khun,* literally “liver blood,” is an extreme and persistent dysphoria that includes grief following interpersonal loss but that may also be a reaction to any deeply disappointing or painful experience (59).

As an example in CAP, several tools for autism spectrum disorder showed lower psychometric properties among immigrant minority groups, non-native speakers, and other cultural minorities from non-Western countries, attesting to a worldwide scarcity of validated and culturally attuned screening and diagnostic tools (17, 60). For example, the Autism Diagnostic Interview-Revised (ADI-R) in a Spanish-speaking population in the U.S. showed lower sensitivity and specificity rates than in the original validation that included only native English speakers (61). The Spanish version of ADI-R may be less valid when the parent and child's Spanish language proficiencies differ; if the parent speaks mostly Spanish and the child speaks mostly English with peers and at school, the ADI-R verbal communication questions may be less valid.

Efforts have been made to make ICD-11 culturally sensitive; however ICD-11, as a nomothetic nosography, is not intended to provide support for individual evaluation (62). The cultural formulation interview of DSM-5 includes interesting idiographic questions on cultural definitions of the problem, and some authors have suggested developing a supplementary module specifically for young children (63, 64). More tools are needed to assess other factors such as interactional aspects within a particular cultural context. The matter is complicated further by the changes that inevitably occur in culture over time, resulting in the need for regular reassessments and revisions of these instruments.

Some researchers have designed culturally pertinent scales: Phan et al. designed an adult psychiatric scale that is derived from Vietnamese idioms and cultural understandings of psychiatric and emotional distress identified in Vietnamese literature and using ethnographic methods (57). Another option is to propose transcultural validations and adaptations of existing scales [e.g., cross-cultural validation of the Positive and Negative Syndrome Scale (65)]. A third option is to intentionally develop tools, from their conception, to be applicable across cultures, such as: the PSYca, a tool designed to screen for psychological difficulties among children aged 6–36 months (66), and the ELAL scale, an instrument designed to assess language skills in any minority language and consequently better diagnose language disorders across various populations (65). Kohrt et al. propose criteria to evaluate the cross-cultural validity of CAP instruments, such as the incorporation of mention of local idioms employed, the structure of response sets, and comparisons with other measurable phenomena (67). Within a health justice framework, it is our ethical responsibility as child psychiatrists to develop tools that are adaptable to the needs of different patient populations.

## Clinical practice: provide pluralistic articulated interventions

In clinical practice, it remains a challenge to implement this plurality of approaches and develop different narratives of a patient's condition that can coexist. Articulating pluralistic care in clinical practice can be promoted through the following axes:

• [I] train clinicians in pluralistic care;
• [II] ensure that these interventions are accessible to patients;
• [III] develop competency in determining the temporal sequence of care: which intervention is prioritized and/or can facilitate other interventions (e.g., family intervention is sometimes necessary before a patient can accept medical treatment, or vice versa);
• [IV] encourage diverse care teams with psychiatrists trained in distinct approaches (for complex patients).

For example, the management of eating disorders requires a pluralistic approach as well as a transdisciplinary care team (68, 69). Autism spectrum disorder is another example of a disorder that is explained with a biological approach, treated with interactional and behavioral treatments, and influenced by important cultural and social factors (16) (see Table 1). Some authors have proposed integrative frameworks that encompass developmental, multidimensional, eco-systemic, and multifactorial approaches and are associated with pluralistic interventions (70).

As an illustration of a pluralistic intervention, we carried out a study on a specific mental health care program for unaccompanied immigrant minors: Multimodal Co-Therapy for Unaccompanied Minors (MUCTUM) (Guessoum et al., *under review*). This monthly consultation brings together a standard biological approach (including pharmacological treatment), an institutional approach (aiming to improve caseworker-youth relationships and communication as well as solving stressful daily life problems), transcultural psychiatry (providing an interpreter who speaks the patient's native language and acts as a bond with the patient's native country, cultural affiliations, family, and culturally meaningful care), and a narrative approach (narration of the youth's story, immigration, and trauma). MUCTUM aims to provide a pluralistic approach through the use of two therapists with different backgrounds (a psychiatrist and a psychologist), further enriched by the participation of an interpreter and the youth's caseworker. MUCTUM is consistent with the idea that pluralism requires close collaboration among psychiatrists, psychologists, and social workers (32). In this study, we hypothesize that following a hierarchy of needs, through a patient-centered and problem-solving approach, could be one axis by which to articulate these interventions. We also hypothesize that a pluralistic approach could be provided through interventions in a single consultation with several care providers with both biological and cultural backgrounds. Implementation research for such approaches is much needed in CAP (71).

## Training: pluralistic competency

Suggestions for training in pluralistic competency:

• [I] Provide a basic pluralistic theoretical training (introduction to biological *and* social approaches *and* how to articulate them in clinical practice) early on in medical school;
• [II] Make sure residents complete internships in both biological psychiatry-oriented and social psychiatry-oriented services;
• [III] Train faculty members in basic clinical and teaching skills for both approaches and promote dialogue among faculty members from different fields;
• [IV] Develop and teach interventions that articulate biological and social approaches;
• [V] Provide a model curriculum for pluralistic articulated training.

Training in social approaches is provided at several universities, such as (trans)cultural competency and structural competency (72, 73). McGill University's medical school curriculum provides training grounded in basic social science perspectives (74). Cultural competency notably includes an awareness of the impact of the clinician's own ethnocultural identity on patients, knowledge of the language and cultural background of groups seen in clinical practice and their interactions with mental health issues and treatment, and skills for working with particular groups (75). Structural competency training relies on developing a capacity to identify how social, economic, and political conditions produce health inequalities and can shape symptoms and diseases (72, 76). After identifying such factors, the clinician would then mobilize to address the inequities in the clinician-patient dynamic or in the patient's life. Transcultural competency developed in France (77), describes the ability of clinicians to provide culturally appropriate care, allowing the co-existence of medical and patients' cultural narratives and treatments. Training in transcultural psychiatry in Université Paris Cité in France is embedded in the early curriculum and provided in later programs for care providers and cultural mediation (73, 78). Training also occurs through mentoring during consultations, lecture courses, role-playing, or use of senior resident-assisted consultations.

Pluralistic competency would call for basic skills in both biological and social approaches, which enables one to identify the most suitable approach for a given patient and refer patients to specialists when necessary, e.g., having basic skills in transcultural psychiatry allows any clinician to seek for a cultural etiology of the disease and evaluate its importance in the patient's and their family's narration of the disorder and the need for specialized transcultural therapy if available. Another central aspect is dialogue, self-esteem, and collaborative work among care providers from different disciplines to achieve a pluralistic approach, including multidisciplinary staff meetings.

## Discussion

This article discusses the articulation and implementation of a pluralistic approach. There is still a lack of appropriate tools, pluralistic syntheses, integrative knowledge, and training for professionals in these approaches. More biological studies should be conducted in non-Western countries to obtain results applicable in these settings and address the population homogeneity needed in many biological studies. Distinct avenues for cross-cultural perspectives allow for the comparison of data from disparate item sets and response formats, such as mega-analyses of aggregated heterogeneous data from many individuals (79).

Promoting the implementation of pluralistic care in psychiatry requires assessing its financial cost. Evidence exists for the economic and social value of specific interventions in CAP (80), e.g., proposing specialized transcultural care, in addition to standard approaches, may be cost-effective for health systems (81).

From an ethical standpoint, the driving force behind such research should be improving the quality of health care, not economic factors only (25, 82, 83). Promoting pluralistic research that includes social factors is necessary to provide just and equitable health care (84). This includes ethically justified exclusion criteria (82). Promoting such approaches requires that they be available and accessible to both patients and professionals, and supporting free online access to diverse tools may be a simple contribution.

Articulating biological and social approaches in CAP in clinical practice and research is an ongoing challenge. As a conclusion, we provide highlights in Table 2.

**Table 2:** Take-home messages for articulating biological and social approaches in child and adolescent psychiatry.

**I. State your preferred epistemology: explain the approach you chose and why**	A research object can be approached from different clinical, research, and theoretical angles. Readers of scientific articles should be aware of the existence of different epistemologies. Authors should state in the introduction that several approaches to the topic are possible and explain the reasoning for their chosen approach.
**II. Write and read articles that articulate biological and social knowledge (case studies, clinical perspectives, literature reviews, etc.)**	To avoid knowledge silos, researchers and clinicians documenting psychiatric phenomena should provide both social and biological data in the same article. This will improve reflection on clinical interventions and research designs by shaping readers’ representations and allowing them to address the complexity of the topic.
**III. Always take culture into account: Design, assess, and implement transcultural tools in research, clinical practice, and teaching.**	Transcultural tools (i.e., validated and usable in various cultural settings) can be an interface between biological and social factors in research design. Transcultural tools are more relevant for real-world clinical practice and the cultural diversity of patients. Standardized assessments using validated transcultural tools lead to more accurate and reliable results, even for neurobiological conditions and mitigate cultural biases.
**IV. Research—Think outside the box: design hybrid studies**	Design biological studies that consider social factors and social science studies that consider biological factors. E.g., a biological study design should consider factors such as culture, language, immigration, socioeconomic status, and life circumstances. E.g., a social science study should consider the disorder, the existence of an underlying neurobiological or organic condition, and the use psychotropic medication during study.
**V. Clinic—Work with diversity: Develop pluralistic interventions that articulate biological and social approaches**	Pluralistic interventions that combine distinct approaches should be designed, implemented, and assessed, with specifications about how they should be implemented, such as the temporal sequence of interventions. The development of diverse care teams can support the implementation of articulated approaches.
**VI. Teaching—Develop curricula in pluralistic articulated care**	Clinicians require training that addresses the existence of diverse epistemologies, establishes an awareness of the need for pluralistic approaches, and develops clinical skills in prescribing and implementing pluralistic approaches.

## Data Availability

The original contributions presented in the study are included in the article/**Supplementary Material**, further inquiries can be directed to the corresponding author/s.
