# Supplementary material for: Articulating biological and social approaches in child and adolescent psychiatry
Source: Front Child Adolesc Psychiatry. 2022 Dec 21;1:1065932. doi: 10.3389/frcha.2022.1065932 (PMC11731969; doi:10.3389/frcha.2022.1065932)
Supplement: Supplementary file 1 [file Table1.docx]

CITATION Guessoum SB, Benoit L, Thomas I, Mallet J, Sibeoni J, Hanin C and Moro MR (2022) Articulating biological and social approaches in child and adolescent psychiatry. Front. Child Adolesc. Psychiatry 1:1065932. doi: 10.3389/frcha.2022.1065932

**Supplementary materials: Brief definitions of some core concepts**

| **A theory** is an idea or set of ideas that is intended to explain facts or events.  **A biological approach** focuses on the biomedical dimension of mental health and is grounded in human biology and neuroscience.  **A social approach** focuses on the social and cultural dimensions of mental health and is grounded in the social sciences and the humanities.  **A pluralistic approach** states that multiple independent methods are necessary in the understanding and treatment of mental illness.  **An integrative approach** aims to provide a synthetic approach to the distinct fields within psychiatric research, diagnosis, and treatment.  **Complementary frames of reference** involves the obligatory but non-simultaneous use of several disciplines.  **Nomothetic** describes an object or method that enables one to establish repeatable and generalizable patterns, is concerned with general laws and theories, and is represented by constant relations between the observed phenomena.  **Idiographic** describes an object or method within social research that focuses on specific elements, individuals, events, entities, situations, documents, and works of art and culture and concentrates on what is particular to these.  **Multimodal** describes a combination of several interventions or treatments.  **A multidisciplinary approach** is based on the knowledge of different disciplines while respecting their delineations.  **An interdisciplinary approach** analyzes, synthesizes, and harmonizes the links between disciplines into a coordinated and cohesive whole.  **A transdisciplinary approach** integrates distinct fields of study and crosses their traditional boundaries.  **A categorical approach** establishes predefined diagnostic categories. Psychiatric diagnosis aims to identify the presence or absence of a psychiatric disorder in a patient and then to classify the patient's disorder into a diagnostic category according to pre-established criteria.  **A dimensional approach** is based on the conception of symptoms as a continuum between normal and pathological and as not being exclusive across diagnostic categories. Psychiatric diagnosis aims to evaluate the different dimensions of the patient's symptoms and to nuance them according to their intensity. |
| --- |

These definitions are a synthesis of the data found in this article’s references and in dictionaries [(13,23,85–88)](https://paperpile.com/c/H3qIUF/kiPOu+u9udU+oLrjJ+dmksh+Cc4rk+lkrWU).
